# A Shift in Sensory Processing that Enables the Developing Human Brain to Discriminate Touch from Pain

**DOI:** 10.1016/j.cub.2011.08.010

**Published:** 2011-09-27

**Authors:** Lorenzo Fabrizi, Rebeccah Slater, Alan Worley, Judith Meek, Stewart Boyd, Sofia Olhede, Maria Fitzgerald

**Affiliations:** 1 Department of Neuroscience, Physiology and Pharmacology, University College London, London WC1E 6BT, UK; 2 Department of Statistical Science, University College London, London WC1E 6BT, UK; 3 Nuffield Department of Anaesthetics, University of Oxford, Oxford OX3 9DU, UK; 4 Department of Clinical Neurophysiology, Great Ormond Street Hospital for Children, London WC1N 3JH, UK; 5 Elizabeth Garrett Anderson Obstetric Wing, University College Hospital, London NW1 2BU, UK

## Abstract

When and how infants begin to discriminate noxious from innocuous stimuli is a fundamental question in neuroscience [1]. However, little is known about the development of the necessary cortical somatosensory functional prerequisites in the intact human brain. Recent studies of developing brain networks have emphasized the importance of transient spontaneous and evoked neuronal bursting activity in the formation of functional circuits [2, 3]. These neuronal bursts are present during development and precede the onset of sensory functions [4, 5]. Their disappearance and the emergence of more adult-like activity are therefore thought to signal the maturation of functional brain circuitry [2, 4]. Here we show the changing patterns of neuronal activity that underlie the onset of nociception and touch discrimination in the preterm infant. We have conducted noninvasive electroencephalogram (EEG) recording of the brain neuronal activity in response to time-locked touches and clinically essential noxious lances of the heel in infants aged 28–45 weeks gestation. We show a transition in brain response following tactile and noxious stimulation from nonspecific, evenly dispersed neuronal bursts to modality-specific, localized, evoked potentials. The results suggest that specific neural circuits necessary for discrimination between touch and nociception emerge from 35–37 weeks gestation in the human brain.

## Highlights

► The human brain may discriminate touch from pain from 35–37 weeks gestation ► Before 35–37 weeks, touch and noxious lance evoke nonspecific neuronal bursts ► After 35–37 weeks, touch and noxious lance evoke modality-specific potentials

## Results

### Characterization of Tactile and Noxious-Specific Brain Activity in Full-Term Infants

**Figure 1:**
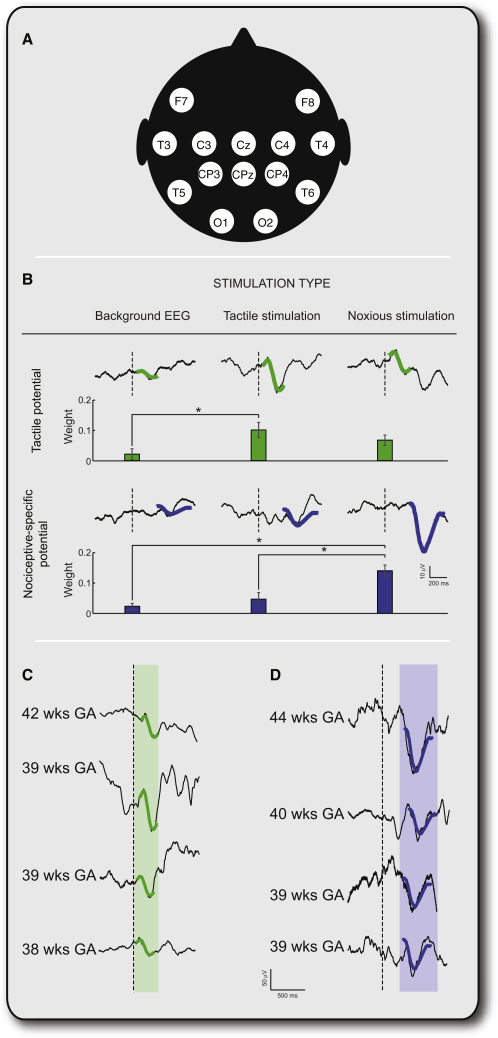
Time-Locked Touch and Noxious Lance of the Heel Evoke Tactile and Nociceptive-Specific Potentials in Full-Term Infants (A) Electrode placements for EEG recordings (modified international 10/20 electrode placement system). (B) Dependence of the principal component (PC) weights on stimulus modality at CPz (mean ± standard error of the mean). The PC obtained between 50 and 300 ms after the stimulus onset represents a tactile potential, and the PC obtained between 300 and 700 ms after the stimulus onset represents a nociceptive-specific potential. The PCs (bold lines) are overlaid on the averages obtained across the three stimulation types (background EEG, touch, noxious lance). (C) Examples are shown here of the tactile potential at CPz evoked by touch in four full-term infants. (D) Examples are shown here of the nociceptive-specific potential at CPz evoked by noxious lance in four full-term infants. The shadowed areas mark the time interval in which the PC analyses were conducted.

To investigate the emergence of specific neural activity evoked by tactile and noxious stimulation in the developing human infant brain, we first defined this activity in full-term infants. Principal component analysis (PCA) was used to identify tactile and nociceptive-specific potentials following time-locked touch or noxious lance of the heel (clinically required for blood samples) of 18 infants, aged 37–45 weeks gestational age (GA) (born at 37–41 weeks GA). The electroencephalogram (EEG) activity, recorded using a modified international 10/20 electrode placement system (Figure 1A), showed that full-term infants display distinct and separable responses to tactile and noxious stimuli recorded at the midline (CPz; Figures 1B–1D), as previously reported [6].

The tactile potential was defined by the first principal component (PC) in the EEG at CPz between 50 and 300 ms after stimulation because the weight of this component was significantly larger following touch compared to the background EEG (one-way analysis of variance [ANOVA]: F_2.66_ = 3.72, p < 0.05; least square difference (LSD) post hoc comparison: p < 0.05). The nociceptive-specific potential was defined by the second PC at CPz between 300 and 700 ms after stimulation because the weight of this component was significantly larger following noxious lance compared to the touch or background EEG (one-way ANOVA: F_2,66_ = 11.97, p < 0.05; LSD post hoc comparison: p < 0.05). These principal components were highly correlated with those defined in a previously published separate sample of 44 term infants [7] (correlation coefficients: 99% [tactile potential]; 95% [nociceptive-specific potential]).

### Age Dependence of Tactile and Nociceptive-Specific Potentials—Comparison between Full-Term and Preterm Infants

Having characterized tactile and nociceptive-specific potentials in full-term infants, we next determined whether such activity was present in younger, preterm infants. The equivalent weights of the PCs representing the tactile and nociceptive-specific potentials in full-term infants were calculated at Cz in 60 EEG epochs (30 in term and 30 in preterm infants) following time-locked touch or noxious lance of the heel of 41 infants, aged 28–45 weeks GA (born at 24–41 weeks GA).

Tactile and noxious-specific potentials were evoked in a small proportion of the preterm infants. The percentage of occurrence in the preterm infant population was significantly less than in the full-term infant population (preterm infants: 7% tactile potential [2/30], 33% nociceptive-specific potential [10/30]; term infants: 30% tactile potential [9/30], 63% nociceptive-specific potential [19/30]) (one-tailed z test: p = 0.01 tactile potential; p = 0.01 nociceptive-specific potential). The latencies of the tactile and nociceptive-specific potentials were shorter in full-term infants than in premature infants (on average 31 ms shorter for the tactile potential; 40 ms for the noxious-specific potential).

The occurrence of the noxious-specific potentials was not influenced by the arousal state of the infants at the time of stimulation. The proportion of awake infants who exhibited the noxious-specific potential (41%; 12/29) was not significantly different (two-tailed z test: p = 0.64) from the proportion of awake infants who did not (35%; 11/31).

### Time-Locked Touch and Noxious Lance of the Heel Evoke a Nonspecific Neuronal Burst in Preterm Infants

**Figure 2:**
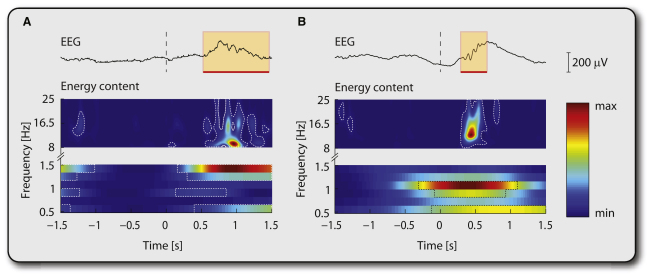
Both Time-Locked Touch and Noxious Lance of the Heel Trigger a Neuronal Burst (A) Example is shown here of a neuronal burst recorded from the temporal region in a preterm infant (34 weeks gestational age [GA]) following time-locked touch of the heel. (B) Example is shown here of a neuronal burst recorded from the temporal region in a different preterm infant (34 weeks GA) following time-locked noxious heel lance. The area shadowed in orange highlights the neuronal burst. Significant changes in signal energy from baseline are delineated by the dashed lines.

Next we measured the occurrence of a neuronal burst in both the preterm and full-term infants using the same infant EEG recordings that were used for analysis of tactile and nociceptive-specific evoked potentials. A neuronal burst (delta brush) was defined here as a significant change from the baseline energy occurring simultaneously in the low frequency band (0.5–1.5 Hz) and the high frequency band (8–25 Hz), recorded at any electrode site within the first 1.5 s after stimulation and displaying characteristic negative polarity, amplitude, and duration [8] (Figure 2).

In preterm infants, the spontaneous occurrence of a neuronal burst was recorded in 13% (4/30) of the background EEG epochs. Both touch and noxious lance increased the rate of occurrence, but this effect was greater following noxious lance (57%; 17/30) than following touch (27%; 8/30).

The same neuronal bursting activity was recorded in 10% (3/30) of background EEG epochs in full-term infants, but an increase in this activity following sensory stimulation of the heel was significantly less common in this group. In contrast to the preterm population, neuronal bursts followed only 10% (3/30) of touch stimuli and 13% (4/30) of noxious lance stimuli in full-term infants. The percentage of occurrence of neuronal bursts was significantly greater in preterm infants than term infants whether evoked by touch (one-tailed z test: p = 0.048) or noxious lance (p < 0.001).

### Maturation of Evoked Nonspecific Neuronal Bursts into Tactile and Nociceptive-Specific Potentials

Touch or noxious lance of the heel are significantly more likely to evoke tactile and noxious-specific potentials in full-term infants but significantly more likely to trigger a neuronal burst (delta brush) in preterm infants. This led us to hypothesize that the nonspecific neuronal bursts triggered by heel stimulation in preterm infants might gradually mature into tactile and nociceptive-specific potentials in the full-term infants. Furthermore, the age at which the change from nonspecific neuronal bursts to specific potentials occurs could be determined by plotting the occurrence of each type of response, across the sample, with respect to gestational age.

**Figure 3:**
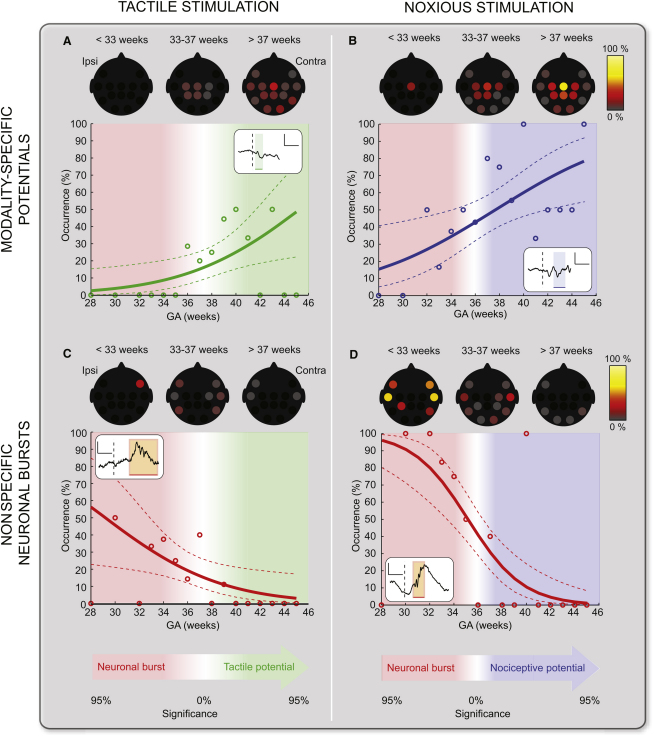
Relationship between Response Type, Nonspecific Neuronal Burst, or Modality-Specific Potentials, Evoked by Tactile and Noxious Stimulation, with Gestational Age Age dependence of the occurrence and topographical distribution of tactile (A), nociceptive-specific potentials (B), and nonspecific neuronal bursts (C and D) following tactile or noxious stimulation of the heel. The occurrence of each type of activity is shown on a week-by-week basis (circles) together with the generalized linear model (GLM) fitted to the data (solid lines) and 90% confidence intervals (dashed lines). An example of each response is illustrated in the inset; the dashed lines represent the time of stimulation and the scale bars represent 100 μV (vertical) and 500 ms (horizontal). At the bottom of the figure, the transition from the neuronal bursts to the modality-specific potentials is described as the significance of the difference between the respective occurrences. Touch and noxious lance were more likely to evoke a tactile and nociceptive-specific potential than a neuronal burst from approximately 35–37 weeks GA.

Figure 3 shows how the occurrence of the two types of activity, nonspecific neuronal bursts and tactile and nociceptive-specific potentials, in response to touch and noxious lance of the heel, gradually change with gestational age. The occurrence of tactile and nociceptive-specific potentials gradually increases with gestational age, mostly at central electrode sites (Figures 3A and 3B). The occurrence of neuronal bursts triggered by the same touch and noxious lance stimuli is mainly at temporal and frontal electrode sites and gradually decreases over the same age period (Figures 3C–3D). The data indicates that there is a critical crossover period in brain development, after which the somatosensory circuitry in the brain has matured sufficiently to produce predominantly specific potentials rather than nonspecific neuronal bursts in response to the same touch or noxious lance stimuli. The critical period is 35–36 weeks GA for nociceptive processing and 36–37 weeks GA for tactile processing; however, these are not significantly different (p = 0.96).

## Discussion

This is the first study to systematically map the maturation of tactile and nociceptive activity in the developing human brain from the extremely preterm stage (28 weeks) through to the age of normal full-term birth (>37 weeks). The aim was to understand how and when the circuitry required for touch and pain discrimination emerges in the human brain. The importance of this study lies not only in deepening our understanding of early human brain somatosensory function but also in the insight it provides about the onset of specific nociceptive processing, most likely required for pain perception, in the central nervous system.

We show here that neural activation by peripheral tactile and noxious stimulation occurs from an early preterm stage but that there is a change in the pattern and specificity of the response with age. At early stages of brain development, less than 35 weeks gestation, the dominant response to both touch and noxious lance of the heel is an increased incidence of nonspecific neuronal bursts. At later stages of brain development, after 35–37 weeks gestation, the dominant response is quite different. Touch and noxious lance of the heel now evoke characteristic somatosensory potentials, maximal at the central electrodes, which are completely separable in timing and morphology for the two modalities of stimulation. Thus, specific somatosensory tactile and nociceptive potentials appear to emerge from a transient, nonspecific neuronal bursting activity.

Synchronized neuronal bursting activity has been reported in numerous immature neuronal circuits in both man and laboratory animals. This endogenous neuronal activity during prenatal and early postnatal brain development is thought to be a key factor in the organization of functional cortical neuronal networks by guiding synapse formation, elimination, and rearrangement [9, 10]. Such transient spontaneous neuronal bursting activity has been recorded with EEG in premature and very young infants at different developmental stages [11–14] and used for clinical prognosis because their persistence at full term or absence during development is correlated with brain abnormality [15–18]. The neuronal bursting activity that we report was defined as delta brushes [11, 13] consisting of fast frequency ripples of 8–25 Hz superimposed on a slow wave of 0.3–1.5 Hz [3, 8, 19, 20]. Delta brushes increase in frequency and amplitude with maturity, becoming most prominent and frequent at 32–34 weeks postmenstrual age (PMA) and decrease by 42 weeks PMA. Before 28 weeks PMA, they are mostly expressed in central areas of the brain, whereas from 28 weeks to near term they spread also to the temporal, frontal, and occipital areas [8]. Our finding that delta brushes were triggered by touching the heel is consistent with previous reports of activation by spontaneous hand and foot movements and tactile stimulation [20]. Delta brushes are similar in terms of developmental profile, frequency characteristic, and topography to the spindle bursts recorded in the cortex of neonatal rats, which are also triggered by sensory stimulation and correlated with spontaneous sleep-related myoclonic twitches through sensory feedback [21, 22]. Here we show that noxious stimulation of the body also triggers the same neuronal bursting activity but with greater incidence than after tactile stimulation. The fact that neuronal bursts can be triggered by a wide range of sensory stimuli across broad areas of the brain [3, 4] suggests that the higher incidence of bursts triggered by heel lance reflects the intensity of the input rather than any specific discrimination of stimulus modality.

The somatosensory evoked potentials that we recorded in older infants were the same as described previously from EEG recording in response to touch and noxious lance of the heel [6, 23]. The characteristics of the tactile potential reported here are similar in morphology and topography to the vertex potentials evoked by electrical stimulation and time-locked tapping of tendons and muscles [24, 25], auditory click [26], and, to a certain extent, to those evoked by visual stimulation in full-term newborns [25, 26]. A similar potential has also been recorded in adults following visual, auditory, nonnoxious, and noxious laser stimulation [27]. Although we have used the term “tactile potential” to describe the potential evoked by both the nonnoxious and noxious stimulation, we do not know what aspect of neuronal processing this potential represents. It may be directly related to the tactile input or reflect a nonmodality-specific process, such as attention or arousal. On the other hand, the characteristics of the infant nociceptive-specific response, which was only evoked in response to the noxious stimulus, are strikingly similar in polarity and topography to cerebral potentials evoked by “painful” mechanical stimulation in adults [28]. The clearly defined specific noxious-evoked potentials recorded in older infants in response to heel lance, in contrast to the nonspecific increase in neuronal bursts characteristic of early prematurity, are therefore likely to reflect the maturation of the functional brain circuitry that enables the human brain to discriminate noxious stimuli from other forms of sensory input.

Neuronal bursts are more evenly distributed across electrode sites than the evoked potentials, as might be expected if the neuronal bursts represent activity in more immature networks across the brain. This activity may contribute to the previously reported hemodynamic activity recorded in the contralateral somatosensory cortex following noxious stimulation from 25 weeks gestation [29]. We do not know which features of the evoked neuronal activity drove the change in total hemoglobin concentration; however, the occurrence of the neuronal bursts shows a slight prevalence on the contralateral side. In contrast, the nociceptive-specific and tactile potentials were maximal at the midline region of the brain, consistent with observation in adult studies [28].

The transition from predominantly nonspecific neuronal bursts to specific evoked potentials occurs at 35–37 weeks gestation, which is just before an infant would normally be born. Several mechanisms have been proposed to underlie neuronal bursting activity in developing circuits, including depolarizing GABA, extrasynaptic glutamate, gap junctions, and transient connections [2]. In the somatosensory cortex, bursting develops in vitro, emphasizing its intrinsic nature, but is strongly modulated by sensory inputs [22] and interhemispheric connections in vivo [30]. The transition from neuronal bursts to evoked potentials in the somatosensory and nociceptive processing at 35–37 weeks gestation in the human brain is consistent with previous reports [25] and is in concurrence with a similar shift in the visual system [4]. The transition in the processing of peripheral stimuli may be related to the structural development of thalamocortical connections and the formation of callosal and association pathways at around this time [31].

Our findings are in line with the idea that neuronal bursts represent the activation of neuronal circuits, which can spontaneously fire or may be triggered by peripheral activation, such as spontaneous retinal waves or somatosensory input that may occur in utero. However, the 35-week-GA human infant would not normally be exposed to noxious stimulation and therefore it is unlikely that noxious sensory input is a requirement for early cortical pain circuit development. A key role may be played by the pacemaker neurons that have been recently discovered within the maturing lamina I of the spinal cord and provide an endogenous drive to the developing pain circuitry [32].

Repeated noxious stimulation of the kind used in this study is a feature of neonatal intensive care [33]. Our finding that noxious heel lance increases neuronal bursting activity in the brain from the earliest age raises the possibility that excess noxious input may disrupt the normal formation of cortical circuits and that this is a mechanism underlying the long-term neurodevelopmental consequences and altered pain behavior in ex-preterm children [34–36]. In the adult, pain is a complex, subjective experience with sensory and affective components involving multiple brain regions [37, 38]. We propose that the transition from nonspecific neuronal bursts to specific evoked potentials is a first stage in the development of central pain processing. The timing of this change marks the functional maturation of cortical circuitry such that the human brain can discriminate noxious sensory input from other nonnoxious sensory stimulation.

## Experimental Procedures

### Subjects

Forty-six infants, recruited from the intensive care unit, special care baby unit, and postnatal ward at the Elizabeth Garrett Anderson and Obstetric Hospital, participated in this study. Three infants were studied on more than one occasion. GA was determined from antenatal ultrasound scans taken at 19–20 weeks gestation or from the maternal report of the last menstrual period. This is equivalent to PMA. Table 1 gives the demographic characterization of the subjects.

Medical charts were reviewed and, at the time of study, infants were assessed as clinically stable. Infants were not eligible for inclusion in the study if they were (1) receiving analgesics, sedatives, or other psychotrophic agents; (2) showing signs of tissue damage on the lower limbs; (3) born to diabetic mothers or opioid users; (4) asphyxiated at birth; or (5) born with congenital malformations or other genetic conditions.

Ethical approval was obtained from the University College Hospital ethics committee, and informed written parental consent was obtained prior to each study. The study conformed to the standards set by the Declaration of Helsinki guidelines.

### EEG Recording

Recording electrodes (disposable Ag/AgCl cup electrodes) were positioned according to the modified international 10/20 electrode placement system at F7, F8, Cz, CPz, C3, C4, CP3, CP4, T3, T4, T5, T6, O1, and O2. A reduced number of electrodes were used if access to the infant was limited. Reference and ground electrodes were placed at FCz and the chest, respectively. The impedance of the electrode-skin interface was kept to a minimum by rubbing the skin with an EEG prepping gel, and conductive EEG paste was used to optimize contact with the electrodes. Electrodes were held in place by an elastic net and leads were tied together to minimize electrical interference. EEG activity, from DC (or 0.05 Hz in eight cases) to 70 Hz, was recorded using the Neuroscan (Scan 4.3) SynAmps2 EEG/EP recording system. Signals were digitized with a sampling rate of 2 kHz and a resolution of 24 bit.

### Experimental Protocol

The noxious stimulus was a clinically required heel lance performed to collect a blood sample. No heel lances were performed solely for the purpose of the study. Tactile stimulation was applied by lightly tapping a tendon hammer against the heel of the infants. The EEG recordings were automatically marked at the time of the stimulus in order to select EEG epochs in correspondence of the events for analysis. Tactile and noxious stimulation were performed on the same foot for each infant.

### EEG Epoch Analysis

The tactile and nociceptive-specific potentials were defined from the EEG recordings at CPz of 18 term infants born at term (GA: mean = 40.58 weeks; range = 37.71–45.28 weeks). 1.7 s EEG epochs, starting 0.6 s before the event, corresponding to noxious heel lances (n = 23), touches of the heel (n = 23), and no stimulation (background EEG, n = 23), were considered. PCA was used to characterize the evoked activity following time-locked touch and lance of the heel (see Supplemental Experimental Procedures available online).

The neuronal bursts (delta brushes) were defined in the EEG recordings at any electrode site of 21 preterm infants (GA: mean = 34.36 weeks; range = 28.43–36.86 weeks). Six-second EEG epochs, starting 3 s before the event, corresponding to noxious heel lances (n = 30), touches of the heel (n = 30), and no stimulation (background EEG, n = 30) were considered. Delta brushes were identified in accordance to their characteristic frequency, polarity, amplitude, and duration (see Supplemental Experimental Procedures).

The occurrence of tactile and nociceptive-specific potentials and of delta brushes was assessed on 60 EEG recordings following time-locked touch or noxious lance of the heel of 41 infants, aged 28–45 weeks GA (born at 24–41 weeks GA). These infants included the 18 term infants born at term and the 21 preterm infants mentioned above (3 of which were also studied at term), plus 2 infants born prematurely and studied at term. Age at birth was not considered as a variable in this analysis. The occurrence of the tactile and nociceptive-specific potentials was evaluated at Cz rather than CPz because it was an independent electrode site not used to define the potentials, where similar tactile and nociceptive-specific potentials were recorded as at CPz (Figure S1) [6]. Statistical independence here refers to properties of the measurement errors.

The maturation of neuronal bursts into tactile and nociceptive-specific potentials was evaluated as changes in their occurrence in respect to each other across infant gestational age.

All the EEG epochs considered in this analysis were baseline corrected by subtracting the mean baseline signal and high-pass filtered at 0.1 Hz (fourth-order bidirectional Butterworth filter).

In 17 test occasions, the heel-lancing procedure was performed twice to collect the required blood sample; for these infants, two epochs corresponding to tactile stimulation and background control EEG were also analyzed. A total of 68 noxious heel lances were analyzed; of these, 3 were excluded from analysis because automatic event marking of the EEG did not occur and 5 because of the presence of movement artifacts in the EEG recording. Epochs corresponding to tactile and background control EEG in these test occasions were also not analyzed.

## Figures and Tables

**Table 1:** Demographic Characterization of the Subjects

Number of infants	46
Number of stimuli (touch or noxious lance of the heel)	68
Mean (SD^a^) GA^a^ at birth (weeks), n = 46	35.1 (5.4); range 24–41.6
Mean (SD^a^) birth weight (g), n = 46	2,404 (1,070); range 540–4,125
Percentage of males, n = 46	60
Number of multiple gestation infants, n = 46	11
Mean (SD^a^) GA^a^ at time of study (weeks), n = 68	37.3 (3.7); range 28.4–45.3
Mean (SD^a^) postnatal age at time of study (days), n = 68	22.2 (25.7); range 0–110
Number of heel lances performed when the infant had IVH^a^ grades 1, n = 68	4
Number of heel lances performed when the infant had previous surgery, n = 68	10

a SD indicates standard deviation; GA indicates gestational age; IVH indicates intraventricular hemorrhage.

## Supplemental Information

Document S1. One Figure and Supplemental Experimental Procedures
