# Supplementary material for: A Shift in Sensory Processing that Enables the Developing Human Brain to Discriminate Touch from Pain
Source: Curr Biol. 2011 Sep 27;21(18):1552–8. doi: 10.1016/j.cub.2011.08.010 (PMC3191265; doi:10.1016/j.cub.2011.08.010)
Supplement: Document S1. One Figure and Supplemental Experimental Procedures [file mmc1.pdf]

Current Biology, Volume *21*

Supplemental Information

A Shift in Sensory Processing

that Enables the Developing Human Brain

to Discriminate Touch from Pain

Lorenzo Fabrizi, Rebeccah Slater, Alan Worley, Judith Meek, Stewart Boyd, Sofia Olhede, and Maria Fitzgerald

Supplemental Inventory

1. Supplemental Figures and Tables

Figure S1, related to Supplemental Experimental Procedures

2. Supplemental Experimental Procedures

3. Supplemental References

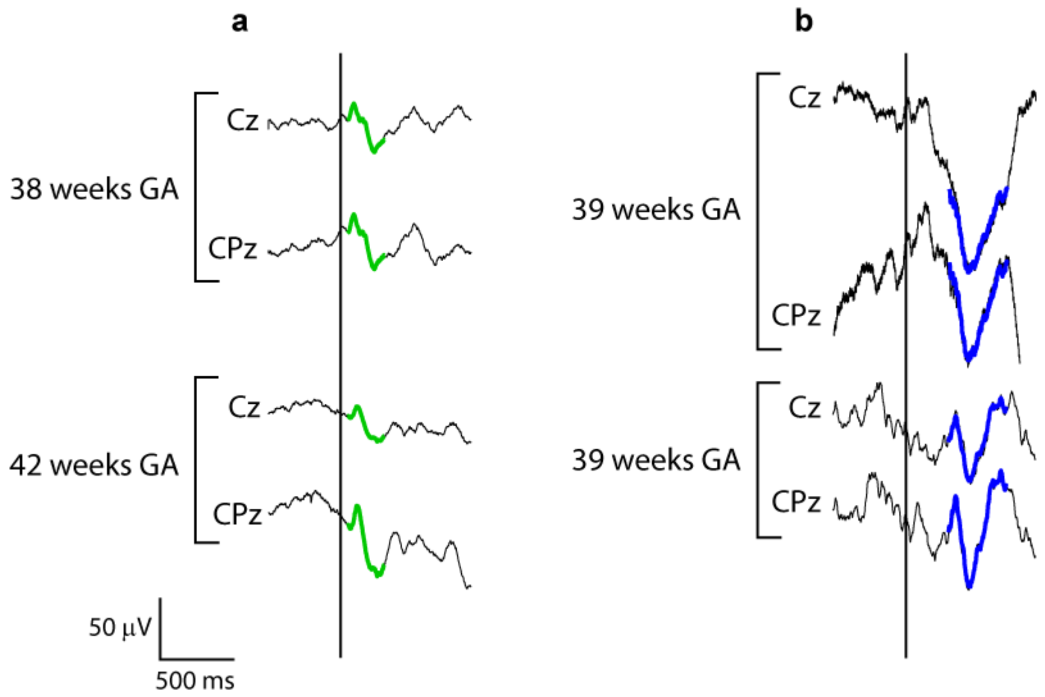

Figure S1. Cz and CPz Display Similar Tactile and Nociceptive-Specific Potentials, Related to Supplemental Experimental Procedures

(A) Examples of tactile potentials evoked at Cz and CPz by time-locked touch of the heel in 2 full term infants (event marked by the black vertical line). In green is the PC calculated for each electrode pair.

(B) Examples of nociceptive-specific potentials evoked at Cz and CPz by time-locked heel lance in 2 full term infants (event marked by the black vertical line). In blue is the PC calculated for each electrode pair.

Supplemental Experimental Procedures

EEG epoch analysis was conducted in the following steps (see Table S1):

1. Tactile and nociceptive-specific potentials were defined in term infants.
2. The dependence of the occurrence of the tactile potential on gestational age was assessed.
3. The dependence of the occurrence of the nociceptive-specific potential on gestational age was assessed.
4. Neuronal bursts were defined in preterm infants.
5. The dependence of the occurrence of neuronal bursts following tactile stimulation on the gestational age was assessed.
6. The dependence of the occurrence of neuronal bursts following noxious stimulation on the gestational age was assessed.

Table S1. Characteristics of Each Analysis Step

Analysis step	Age groups (weeks GA)		EEG epoch (sec)	n of epochs	Stimulation types	electrodes	Technique
	At birth	At test					
1	> 37	> 37	-0.5 to 1.2	23	a,b,c	CPz	PCA
2	24-41	28-45	-0.5 to 1.2	60	a	Cz	Projection on defined PCs + GLM
3	24-41	28-45	-0.5 to 1.2	60	b	Cz	Projection on defined PCs + GLM
4	24-37	28-37	-3 to 3	30	a,b,c	all	Time-frequency analysis
5	24-41	28-45	-3 to 3	60	a	all	Time-frequency analysis + GLM
6	24-41	28-45	-3 to 3	60	b	all	Time-frequency analysis + GLM

Stimulation types: (a) time-locked touch of the heel; (b) time-locked noxious heel lance; (c) background EEG. PCA: Principal Component Analysis; PCs: Principal Components; GLM: Generalised Linear Model. EEG epoch is relative to stimulation onset.

Principal Component Analysis (PCA)

Principal component analysis (PCA) was used to characterise the potentials evoked by time-locked touch and lance of the heel in full term infants. Epochs were considered as variables and time points as observations; the resultant covariance matrix was selected as the association matrix [1, 2]. PCA was performed in two time intervals after traces were aligned to correct for latency jitter [3, 4]: (1) 50-300ms post stimulation (maximum allowed: -50 to +50ms) and (2) 300-700ms post stimulation (maximum allowed: -50 to +100ms). Alignment was obtained by maximising the normalised inner product of the individual epochs with a reference epoch. The reference epoch was the grand average of all the epochs computed iteratively after successive latency adjustments.

One-way analysis of variance (ANOVA) was conducted on the weights of each of the first 2 PCs to determine the effect of stimulation type. The between-subjects effect on the calculated PC weights was negligible ($p>0.05$) compared to the between-trials effect, therefore weights obtained from trials repeated on the same subjects could be included in the ANOVA as independent observations (Appendix A). When the results of the ANOVA were significant

(p<0.05) the group mean differences were tested using least square difference (LSD) posthoc comparisons (p<0.05).

The tactile potential was defined as the PC whose weights were significantly greater following touch of the heel compared to background EEG. The nociceptive-specific potential was defined as the PC whose weights were significantly greater following noxious heel lance compared to touch and background EEG.

Projection on Defined PCs

After the tactile and nociceptive-specific potentials were defined at electrode site CPz in a group of full term infants, the dependence of the weights of their representative principal components with respect to the gestational age at test was assessed.

The equivalent weights of the PCs were calculated for EEG epochs recorded at electrode site Cz. Infants from 28 to 45 weeks GA were considered. The epochs recorded at Cz were not previously used to define the potentials in the full term population.

The following analysis was conducted separately for each PC:

1. Each EEG epoch was filtered between 0.5 and 8 Hz, as this was the frequency band which contained most of the PC energy (applies only to the nociceptive-specific PC).
2. Each EEG epoch was aligned to the PC by maximising the normalized inner product between the epoch and the PC in the time interval were the PC was identified in term infants.
3. The equivalent weights of the PC were calculated for each EEG epoch (Appendix B).
4. The linear least squares regression of the equivalent weights on the gestational age at the time of study was conducted.

Time-Frequency Analysis

Event-related neuronal bursts (delta brushes) were identified in accordance with their characteristic frequencies, polarity, amplitude and duration.

The epochs of EEG signal *s(t)* recorded at each electrode in each trial were convoluted with the complex Morse wavelets, denoted $\psi(t)$. The wavelet $\psi(t)$ can be considered local to a time point and a given scale or frequency. By defining a family of functions $\{\psi_{a,\tau}(t)\}$ by translating and scaling the wavelet function, signal behaviour associated with those times (τ) and scales (*a*) can be isolated by calculating the wavelet transform:

$$W(\tau, a) = \int_{-\infty}^{\infty} \psi_{a,\tau}^{*}(t) s(t) dt \tag{1}$$

Often it is easier to analyse $\{\psi_{a,\tau}(t)\}$, rather than *s(t)*, and by using a complex-valued wavelet, problems with misalignment in phase between the signal and wavelet are avoided. The set of scales and temporal shifts need to be sampled, and the wavelet coefficients were calculated at each observed time point τ and at scales *a* corresponding to pseudo-frequencies between 0.04 and 31.17 Hz in steps of 0.21 Hz. This covered the frequency ranges that define event-related neuronal bursts (delta brushes). A pseudo-frequency is defined by:

$$f_a = \frac{f_c}{a\Delta t} \tag{2}$$

Where f_a is the pseudo-frequency corresponding to scale a, in Hz, Δt is the sampling period of the recording (= 0.5 ms), and f_c is the centre frequency of the Morse wavelet in Hz (= 0.23 Hz). From the wavelet transform the scalogram of the signal at each electrode is calculated by:

$$E(\tau, a) = |W(\tau, a)|^2 = Re(W(\tau, a))^2 + Im(W(\tau, a))^2 \quad (3)$$

We can consider $E(\tau, a)$ to be a measure of the "energy" of the signal at time point τ and pseudo-frequency f_a. To avoid issues with wrap-around at the edges of the epoch, a sub-epoch of 3 seconds starting 1.5 seconds before the event mark was considered for analysis. A neuronal burst (delta brush) was then identified in correspondence to a significant change from the baseline energy occurring simultaneously in at least one frequency f_a belonging to the low frequency band (0.5-1.5 Hz) and at least one frequency f_a belonging to the high frequency band (8-25 Hz) characteristic of the neuronal burst. The time interval when this occurred is successively named T_δ.

The signal $s(t)$ was low-pass filtered at 2 Hz (4th order bidirectional Butterworth filter) and its 1st order derivative $s'(t)$ and 2nd order derivative $s''(t)$ calculated using Richardson's five points formula [5].

Delta brushes are negative deflections therefore:

- *onset* was defined as the time point t_{on} preceding T_δ for which $s'(t_{on}-\Delta t)>0$ and $s'(t_{on}+\Delta t)<0$, or for which $s'(t_{on})<0$ and $s''(t_{on}-\Delta t)>0$ and $s''(t_{on}+\Delta t)<0$;

- *trough* was defined as the time point t_p following t_{on} for which $s'(t_p-\Delta t)<0$ and $s'(t_p+\Delta t)>0$;

- *offset* was defined as the time point t_{off} following t_p for which $s'(t_{off}-\Delta t)>0$ and $s'(t_{off}+\Delta t)<0$ or for which $s'(t_{off})>0$ and $s''(t_{on}-\Delta t)<0$ and $s''(t_{on}+\Delta t)>0$;

The characteristics considered in defining a neuronal burst (delta brush) and their limit values are summarized in Table 2.

Table 2. Characteristics of a Neuronal Burst (Delta Brush)

Characteristic	Calculation	min	max
Amplitude	$A = s(t_{on}) - s(t_p)$	80 μV	300 μV
Duration	$T = t_{off} - t_{on}$	0.3 s	3 s
Duration of T_δ	T_δ	$(t_p - t_{on})/2$	T
Energy ratio of s' in the ascending and descending phase	$R = \frac{\sum_{t_{on}}^{t_p} s'(t)^2}{\sum_{t_p}^{t_{off}} s'(t)^2}$	$\frac{1}{2.5}$	2.5

Generalized Linear Model (GLM)

The proportional occurrence of the tactile and nociceptive-specific potential at Cz (Appendix C) and the proportional occurrence of neuronal bursts at each electrode site was computed at each week of gestational age. Because the proportional occurrences are renormalized binomial random variables, a generalised linear model (with a canonical logit link function) was used to estimate the relationship between occurrence and gestational age at the time of study [6]:

$$\theta(t) = \frac{1}{1 + e^{-(\beta_0 + \beta_1 t)}} \quad (4)$$

The significance of the difference between the occurrence of the neuronal burst and the modality-specific potentials across gestational age was tested as follows.

Considering the GLM of the occurrence of the neuronal bursts following tactile or noxious stimulation:

$$\theta_1(t) = \frac{1}{1 + e^{-(\beta_{10}+\beta_{11}t)}} \quad (5)$$

And the GLM of the occurrence of the tactile or noxious-specific potential:

$$\theta_2(t) = \frac{1}{1 + e^{-(\beta_{20}+\beta_{21}t)}} \quad (6)$$

If we want to look at the difference in occurrence between, for example, the neuronal burst following tactile stimulation and the tactile potential, for any value of *t* we can estimate:

$$\hat{\zeta}(t) = \hat{\theta}_1(t) - \hat{\theta}_2(t) = \hat{\beta}_{10} + \hat{\beta}_{11}t - \hat{\beta}_{20} - \hat{\beta}_{21}t \quad (7)$$

And

$$var\left(\hat{\zeta}(t)\right) = var(\hat{\beta}_{10} + \hat{\beta}_{11}t) + var(\hat{\beta}_{20} + \hat{\beta}_{21}t) =$$
$$= var(\hat{\beta}_{10}) + t^2 var(\hat{\beta}_{11}) + 2cov(\hat{\beta}_{10}, \hat{\beta}_{11})t + var(\hat{\beta}_{20}) + t^2 var(\hat{\beta}_{21}) + 2cov(\hat{\beta}_{20}, \hat{\beta}_{21})t \quad (8)$$

We therefore have that:

$$\hat{\zeta}(t) \sim N(\zeta(t), var\left(\hat{\zeta}(t)\right) \quad (9)$$

So we can compare the estimated difference with the variance to evaluate its significance.

Appendix A. Comparison of Trial Effect and Subject Effect on the PC Weights

The observed weights of each principal component are modelled as:

$$\hat{a}_{ij} = \mu(t_{ij}) + \varepsilon_i + \eta_j \sim N(\mu(t_{ij}), \sigma^2)$$

where *i* denotes the trial and *j* the infant. η_j quantifies the subject effect and ε_i the trial effect and are independent effects. In general, ignoring that the same subjects have undergone multiple trials may result in underestimation of the variance. We define the variance due to the trial effect and to subject effect as:

$$var(\varepsilon_i) = \sigma_\epsilon^2 \quad var(\eta_j) = \sigma_\eta^2,$$

so that:

$$\sigma^2 = \sigma_\epsilon^2 + \sigma_\eta^2$$

If the subject effect is negligible compared to the trial effect ($\frac{\hat{\sigma}_\eta^2}{\hat{\sigma}_\epsilon^2} \approx 0$), then this assumption corresponds to:

$$\sigma_\epsilon^2 \gg \sigma_\eta^2$$

To be able to test this hypothesis a suitable test statistic is needed. Defining I_j the set of trials belonging to the same subject, I_j can have one or two elements. If it has two, then $I_j = \{i(j), k(j)\}$. Under $H_0: \sigma_\eta^2 = 0$, and using $t_{ij} = t_{kj}$:

$$\hat{a}_{i(j)j} - \hat{a}_{k(j)j} = \mu(t_{ij}) + \varepsilon_{i(j)} + \eta_j - (\mu(t_{kj}) + \varepsilon_{k(j)} + \eta_j) = \varepsilon_{i(j)} - \varepsilon_{k(j)} \sim N(0, 2\sigma_\epsilon^2)$$

So:

$$\hat{\sigma}_\epsilon^2 = \frac{1}{2}\left(\frac{\sum_j (\varepsilon_{i(j)} - \varepsilon_{k(j)})^2}{N}\right) = \frac{1}{2}\left(\frac{\sum_j (\hat{a}_{i(j)j} - \hat{a}_{k(j)j})^2}{N}\right)$$

Where *N* is the number of subjects which had repeated trials.

The statistic we need to consider is:

$$T = \frac{\hat{\sigma}_\epsilon^2 + \hat{\sigma}_\eta^2}{\hat{\sigma}_\epsilon^2} = \frac{\hat{\sigma}^2}{\hat{\sigma}_\epsilon^2} \sim F_{a,b}$$

Where *a* are the degree of freedom of the model of the weights and *b* is equal to *N*.

If the subject effect is negligible, then $\hat{\sigma}_\eta^2 \to 0$, therefore *T* has to be below a defined critical value of the $F_{a,b}$ distribution, which corresponds to the null hypothesis holding.

Appendix B. Calculation of the PC Weights of Trials Not Used in the PCA

Consider a data matrix *X* with *n* rows and *m* columns, with *n*<*m*, where the rows are the variables (trials) and the columns are the observations (time points). Removing the mean of the observations from *X* the covariance matrix can be estimated as:

$$C = \frac{1}{m} X X^T$$

Using the singular value decomposition we can represent the covariance matrix as:

$$C = \frac{1}{m} X X^T = \frac{1}{m} U \Gamma V^T (U \Gamma V^T)^T = \frac{1}{m} U \Gamma V^T V \Gamma U^T = U \tilde{\Gamma} U^T$$

With $\tilde{\Gamma} = \frac{1}{m} \Gamma^2$. Therefore the right hand singular vectors *U* of the data matrix are the weights of the PCs.

Consider now the data matrix X_0 recorded from a reference set of trials (term infants) and its singular value decomposition:

$$X_0 = U_0 \Gamma_0 V_0^T$$

The weights U_1 corresponding to another data matrix X_1 associated with the PCs of X_0 can be calculated as:

$$U_1 = X_1 V_0 \Gamma_0^{-1}$$

Appendix C. Test for the Presence of a PC at a Given Gestational Age

According to the linear model the weights of a PC can be modelled as:

$$\hat{a}_j = \beta_0 + \beta_1 t_j + \varepsilon_j$$

where *j* denotes the infant. Then:

$$P(\hat{a}_j > 0) = P\left(\frac{\hat{a}_j - a_j}{\sigma_\varepsilon} > -\frac{a_j}{\sigma_\varepsilon}\right) = 1 - \Phi\left(-\frac{a_j}{\sigma_\varepsilon}\right) = \Phi\left(\frac{a_j}{\sigma_\varepsilon}\right)$$

Where $var(\varepsilon_j) = \sigma_\epsilon^2$. Then considering a significance level α, the null hypothesis is rejected if:

$$\Phi\left(\frac{a_j}{\sigma_\varepsilon}\right) > \alpha \Rightarrow a_j > \Phi^{-1}(\alpha)\sigma_\varepsilon$$

$\alpha = 0.1$ was considered.

Supplemental References

1. Slater, R., Fabrizi, L., Worley, A., Meek, J., Boyd, S., and Fitzgerald, M. (2010). Premature infants display increased noxious-evoked neuronal activity in the brain compared to healthy age-matched term-born infants. Neuroimage *52*, 583-589.
2. Slater, R., Worley, A., Fabrizi, L., Roberts, S., Meek, J., Boyd, S., and Fitzgerald, M. (2010). Evoked potentials generated by noxious stimulation in the human infant brain. Eur J Pain *14*, 321-326.
3. Bromm, B., and Scharein, E. (1982). Principal component analysis of pain-related cerebral potentials to mechanical and electrical stimulation in man. Electroencephalogr Clin Neurophysiol *53*, 94-103.
4. Woody, C.D. (1967). Characterization of an Adaptive Filter for Analysis of Variable Latency Neuroelectric Signals. Med Biol Eng *5*, 539-&.
5. Burden, R.L., and Faires, J.D. (2000). Numerical analysis, 7th Edition, (Brooks Cole).
6. McCullagh, P., and Nelder, J.A. (1989). Generalized linear model, 2nd Edition, (Chapman and Hall/CRC).
